# Supplementary material for: Pilot Study on Exhaled Breath Analysis for a Healthy Adult Population in Hawaii
Source: Molecules. 2021 Jun 18;26(12):3726. doi: 10.3390/molecules26123726 (PMC8234827; doi:10.3390/molecules26123726)
Supplement: Supplementary file 1 [file molecules-26-03726-s001.zip › molecules-1202253-supplementary.pdf]

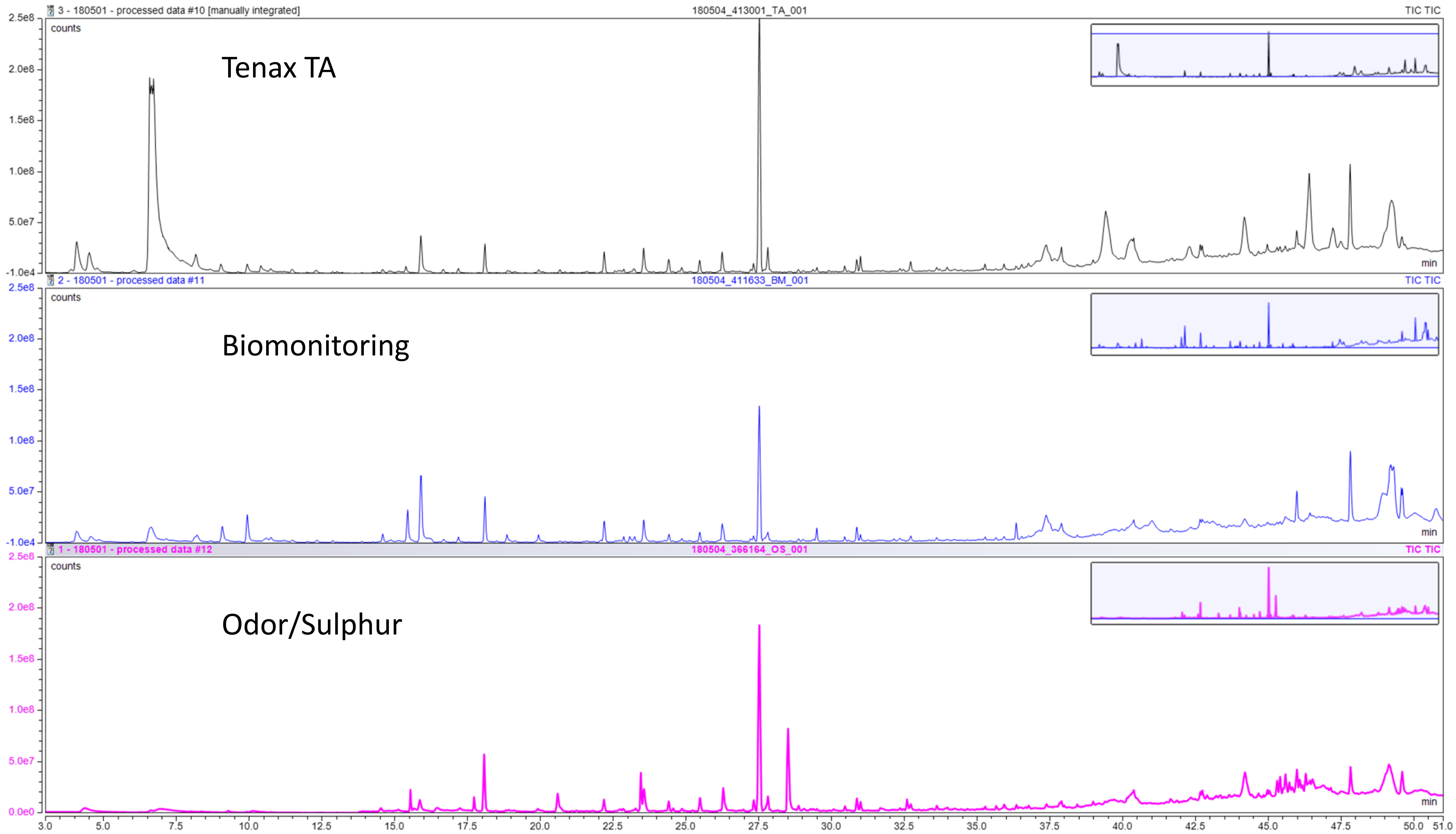

Figure S1. Total ion current chromatograms of Subject 1 on each tube type.

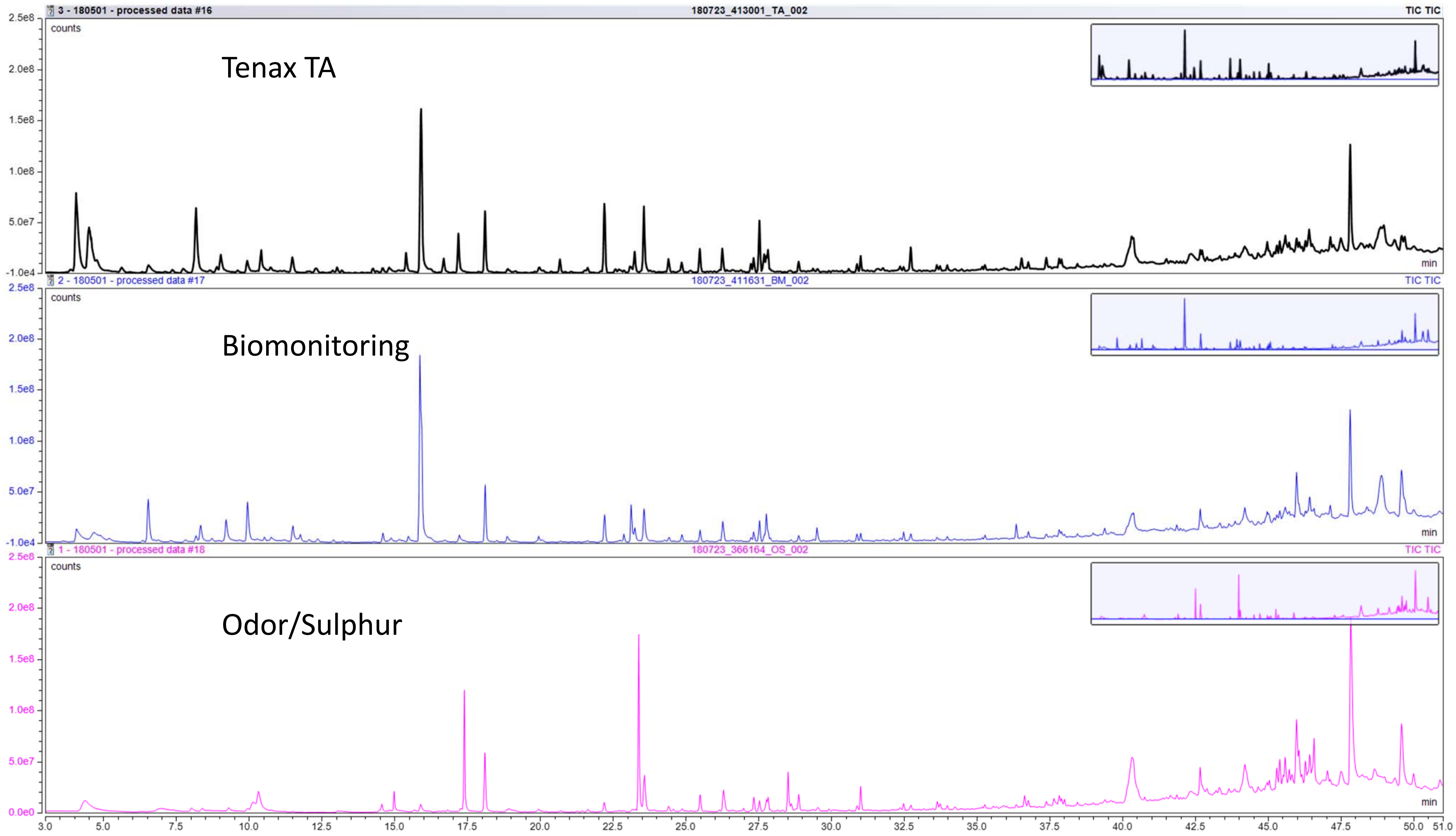

Figure S2. Total ion current chromatograms of Subject 2 on each tube type.

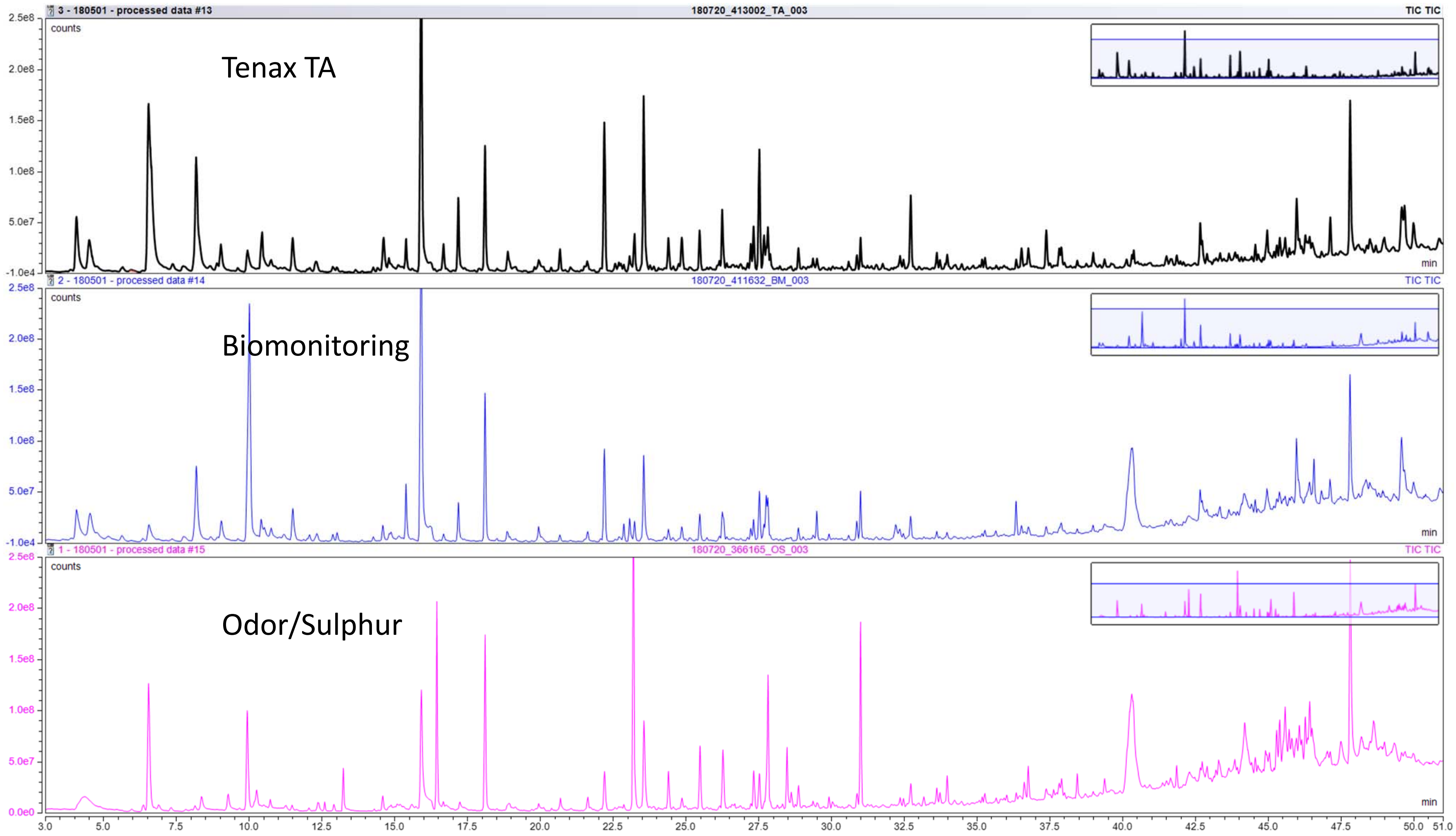

Figure S3. Total ion current chromatograms of Subject 3 on each tube type.
